# A Varactor-Based Very Compact Tunable Filter with Wide Tuning Range for 4G and Sub-6 GHz 5G Communications

**DOI:** 10.3390/s20164538

**Published:** 2020-08-13

**Authors:** Yasir I. A. Al-Yasir, Naser Ojaroudi Parchin, Yuxiang Tu, Ahmed M. Abdulkhaleq, Issa T. E. Elfergani, Jonathan Rodriguez, Raed A. Abd-Alhameed

**Affiliations:** 1 Faculty of Engineering and Informatics, University of Bradford, Bradford BD7 1DP, UK; N.OjaroudiParchin@bradford.ac.uk (N.O.P.); ytu95@yahoo.com (Y.T.); A.Abd@sarastech.co.uk (A.M.A.); R.A.A.Abd@bradford.ac.uk (R.A.A.-A.); 2 SARAS Technology Limited, Leeds LS12 4NQ, UK; 3 Instituto de Telecomunicações, Campus Universitário de Santiago, 3810-193 Aveiro, Portugal; I.t.e.elfergani@av.it.pt (I.T.E.E.); Jonathan@av.it.pt (J.R.); 4 Department of Communication and Informatics Engineering, Basra University College of Science and Technology, Basra 61004, Iraq

**Keywords:** fourth-generation, fifth-generation, sub-6 GHz, computer simulation technology, reconfigurable filter, SPICE, hybrid

## Abstract

A very compact microstrip reconfigurable filter for fourth-generation (4G) and sub-6 GHz fifth-generation (5G) systems using a new hybrid co-simulation method is presented in this manuscript. The basic microstrip design uses three coupled line resonators with λ/4 open-circuited stubs. The coupling coefficients between the adjacent and non-adjacent resonators are used to tune the filter at the required center frequency to cover the frequency range from 2.5 to 3.8 GHz. The coupling coefficient factors between the adjacent resonators are adjusted to control and achieve the required bandwidth, while the input and output external quality factors are adjusted to ensure maximum power transfer between the input and output ports. Two varactor diodes and biasing circuit components are selected and designed to meet the targeted performance for the tunable filter. The impedance bandwidth is maintained between 95 and 115 MHz with measured return losses of more than 17 dB and measured insertion loss of less than 1 dB. Computer simulation technology (CST) is utilized to design and optimize the presented reconfigurable filter, with hybrid co-simulation technique, using both CST microwave studio (MWS) and CST design studio (DS), is applied to build the model by considering the SPICE representation for the varactor switches and all electronic elements of the biasing circuit. The introduced reconfigurable microstrip filter is also fabricated using a Rogers RO3010 material with a relative dielectric constant of 10.1 and it is printed on a very compact size of 13 × 8 × 0.81 mm^3^. An excellent agreement is obtained between the simulation and measurement performance.

## 1. Introduction

Radiofrequency (RF) interference is a big issue in current and future wireless systems such as green RF and wide-band applications, therefore extensive research works have focused on microwave (MW) filters in the last decade [1,2,3,4,5,6,7,8,9]. As part of MW filters, microstrip (planar) filters also play an important role in RF front-end systems of the current and future wireless applications because of size, cost, weight and hardware realization benefits. In order to obtain different characteristics, several planar filters with specific performance have been studied and implemented, such as low-pass filters (LPFs) [10], bandpass filters (BPFs) [11], wideband BPFs [12,13], high-pass filters (HPFs) [14], balanced BPFs [15,16,17], band-stop filters [18] and multi-band BPFs [19,20]. 

Microstrip BPFs are commonly applied to reject unwanted interference signals in several applications, mainly in RF wireless communications due to their main feature to suppress the harmonic frequencies [21,22,23,24]. Recently, the low bandwidth at 700 MHz, the mid bandwidth (3.4–3.8 GHz), and the upper bandwidth (24.25–27.5 GHz) for millimeter-wave are being identified for fifth-generation (5G) spectrum by the office of communications (Ofcom) [25]. Microstrip BPFs are applied to reduce the interference signals in many 4G and 5G applications [26,27,28,29,30,31,32,33,34,35,36,37,38,39]. For microstrip BPFs, the number of poles and zeros, input and output external quality factors, coupling coefficients and the configuration of the resonators are vital features to define the filter performance [40]. Most microstrip filter miniaturization approaches aim to analyze, control or optimize these parameters [41,42,43,44,45]. Besides, several design techniques have been introduced in the literature, such as stepped impedance resonator (SIR) filters, combline filters, open-ring filters, coupled-line filters, and stub impedance filters [46,47,48,49,50,51].

Reconfigurable/tunable RF components have become exciting topics for many researchers and design engineers in recent years [52,53,54]. To reduce the size of the entire wireless application system and tackle miniaturized RF front-ends with better performance, many pieces of research have been carried out for reconfigurable structures and several microstrip tunable BPFs have been introduced in recent years [55,56,57,58,59,60,61,62,63,64]. A microstrip reconfigurable BPF using a varactor diode was designed and analyzed to achieve a maintained impedance bandwidth in [56]. Reconfigurability was obtained by tuning the resonance frequencies for both the odd- and even modes where there is no mutual coupling between these two modes. The practical BPF performance depicted a good roll-off skirt on the low edge of the transmission band with an insertion loss better than 2.2 dB and a return loss of more than 10 dB. A 2.2–22.0 V reverse biasing voltage was applied across the varactor diode to achieve a tuning range of 40% from 0.60 to 1.0 GHz with 91 MHz bandwidth throughout all the configurations.

In [57], a microstrip-reconfigurable BPF utilizes two varactors to tune two finite transmission zeros (TZs) was presented. The center frequency and the bandwidth were controlled to cover a wide range of about 600 MHz (1.4 to 2.0 GHz) by altering the reverse bias voltage across the varactors. The measurement results showed that the filter has an insertion loss of less than 4 dB, a return loss of more than 18 dB, and a fractional bandwidth of about 10%. A stopband rejection level of more than 25 dB was obtained by applying two transmission zeros. A 0.21–30.02 V biasing voltage was applied across reverse biased diodes to tune the resonance frequency with about 600 MHz (1.3–1.9 GHz). In [58], a compact tunable planar BPF with a maintained fractional bandwidth was introduced. By increasing the reverse biasing voltage across the switches, the center frequency of the filter was tuned from 3.4 to 3.8 GHz, with a fractional bandwidth of about 11%. The presented tunable filter has the advantages of the compact size and simple structure, and only one varactor diode switch was applied.

Ebrahimi et al. [59] proposed a notch dual-mode tunable band-stop planar filter using two varactor diodes. The proposed filter was implemented by utilizing inductive and capacitive coupling into the input and output transmission lines of the microstrip filter. The inductors were designed by using thin inductive strips. The second-order filter has a compact size of 0.13 × 0.17λ_g_ and offers a continuous tuning range for the resonance frequency from 0.8 to 1.1 GHz with a stopband fractional bandwidth of about 17%. The measurement results showed that the filter has a 0.9 dB stopband return loss and 0.6 dB passband insertion loss over the entire tuning range. Apart from the other designs, the inductive coupling was achieved using an inductor in the bottom layer of the patch filter. This configuration eliminates the need for a more complicated three-layered structure, provides more degree of freedom in controlling the coupling coefficient factors, and maintains the top layer patch, resulting in a more compact design.

Chen et al. [60] introduced a two-pole fully tunable planar filter with a small structure, continuous frequency tuning range, and constant impedance bandwidth. Two varactors were utilized to tune the resonance frequency between the high and low resonating modes. The tunable filter has a simple configuration that consists of a pair of reversed biased varactor diodes and each resonator designed by two transmission lines which are joined by a varactor diode. A 0.4–18 V biasing voltage was applied to control the varactor diodes and provide 0.3–2.4 pF capacitor. The tuning range for the resonance frequency was from 1.2 to 1.9 GHz with an operational impedance bandwidth of about 39 MHz. The proposed filter offers a compact size of 0.06 × 0.27 λ_g_, continuous tunability, simple structure, and wide-tuned range which makes the designed BPF suitable for recent wireless communications.

Fu-Chang et al. [61] presented a reconfigurable dual-band bandpass-to-bandstop microstrip filter using two sets of half-wavelength resonators integrated with three PIN-diodes and four varactors. The presented filter was printed on s Roger substrate with a dielectric constant of 2.56, loss tangent of 0.003 and height of 0.8 mm, and has a compact area of 36 × 35 mm^2^. The center frequency of the filter is 2.45 GHz, with an impedance bandwidth of about 40 MHz. The center frequency was tuned from 1.7 to 2.9 GHz (26% tuning range) using seven switches, with a return loss of more than 13 dB and insertion loss of about 4 dB.

Moreover, Di et al. [62] proposed a reconfigurable dual-/single-band filter formed by new synchronously controlled dual-mode transmission lines and using four Skyworks SMV1281-079LF varactor diode switches. The selectivity and out-of-band performance have been improved by generating three finite transmission zeros with a rejection bandwidth of up to 3.8 of the center frequency. The center frequency was tuned between 0.76 to 2 GHz, with a changeable impedance bandwidth from 75 to 150 MHz. The insertion and return losses were 1.2 and 15 dB at the center of each band. The designed filter was printed on Rogers RT/droid 5880 substrates with a size of 100 × 8 × 0.5 mm^3^.

However, with the rapid development of current 4G and 5G applications, compact and reconfigurable planar filters with a wide tuning range are needed. To this end, a reconfigurable open-ended bandpass filter with a very compact size and a wide tuning range for fourth-generation (4G) and sub-6 GHz fifth-generation (5G) applications is proposed in this manuscript. Furthermore, a new hybrid co-simulation technique between computer simulation technology (CST) microwave studio (MWS) and CST design studio (DS) is utilized in this structure [65]. The filter is fabricated using a Rogers RO3010 material with a relative dielectric constant of 10.2 and printed on a very compact size of 13 × 8 × 0.80 mm^3^. Altering the reverse biasing voltage across the two switches, a tunable center frequency ranges from 2.5 to 3.8 GHz, with 95–115 MHz impedance bandwidth. The presented design can be updated and combined with the patch antennas [66,67,68,69] to obtain the so-called filtering-antenna structure [70,71,72,73,74]. The presented reconfigurable filter, with the design steps and the obtained characteristics, is detailed in the next sections.

This manuscript is organized as follows. Section 2 discusses the design, analysis and performance of a microstrip asymmetrical coupled line BPF. In Section 3, a new hybrid co-simulation technique is used to design the proposed varactor-based very compact reconfigurable filter with a wide tuning range for 4G and Sub-6 GHz 5G front-ends. Section 4 presents the performance analysis of the proposed reconfigurable filter including the s-parameter, group delay and phase achieved. The performance comparison between the proposed filter with similar designs is also discussed in this section. Finally, Section 5 summarizes the conclusions of our research paper.

## 2. Microstrip Asymmetrical Coupled Line BPF

**Figure 1:**
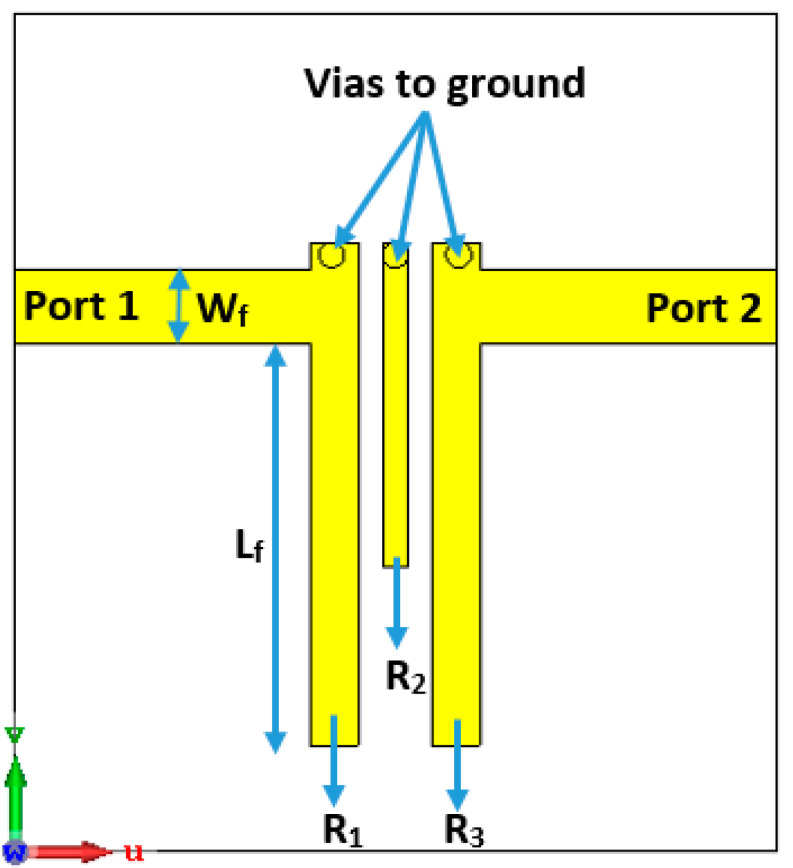
The geometry of the presented three-pole planar bandpass filters (BPF).

The basic structure of the introduced microstrip coupled line BPF is illustrated in Figure 1. The BPF is formed by three-pole transmission line resonators (R_1_, R_2_ and R_3_) and is excited by two ports of 50 Ω impedance. The resonance frequency of 3.7 GHz is selected to design the filter, since it is suitable for sub-6 GHz 5G applications. The coupled lines are open-circuited at one end and short-circuited by vias at the other end. The filter is designed based on a λ_0_/4 resonator, where λ_0_ represents the free-space wavelength corresponding to the 3.7 GHz resonant frequency. The proposed BPF is implemented by using Rogers RO3010 material, with h = 0.80 mm, ε*_r_* = 10.2 and loss tangent = 0.0022. The geometry of the asymmetrical coupled line BPF is illustrated in Figure 1.

**Figure 2:**
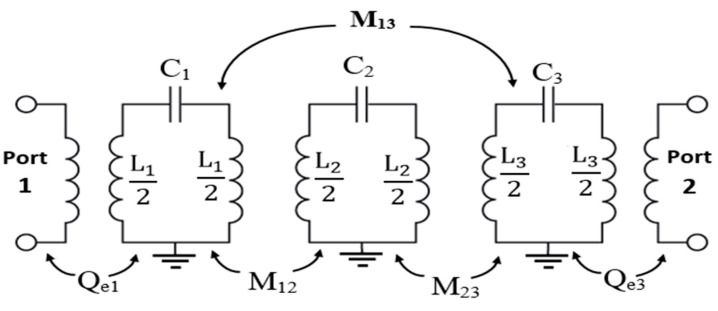
L-C equivalent circuit model of the presented three-pole filter.

The lumped elements equivalent circuit of the 3-pole BPF is illustrated in Figure 2, where, C_1_L_1_, C_2_L_2_ and C_3_L_3_ represent the LC lumped-elements for the resonators R_1_, R_2_, and R_3_, respectively. M_12_ denotes the coupling coefficient factors between the adjacent resonators R_1_ and R_2_, while M_23_ denotes the coupling coefficient between the adjacent transmission lines R_2_ and R_3_. The mutual coupling factor between the non-adjacent transmission lines R_1_ and R_3_ is denoted by M_13_. The external quality factors for the input and output ports are indicated by Q_ei_ and Q_eo_, respectively. The angular frequency of the λ_0_/4 resonator (n) is $\omega_{0n}=2\pi f_{0n}=1/\surd \left(L_{n}C_{n}\right)$, for n = 1, 2 and 3. To simplify the analysis, and since the geometry is symmetric, we can assume that M_12_ = M_23_, Q_e1_ = Q_e3_ and $\omega_{01}=\omega_{03}$. For the introduced BPF, it can be noticed that the cross-coupling between the resonators R_1_ and R_3_ is positive (M_13_ > 0), and this provides that the attenuation poles of finite frequency are on the upper band of the transmission passband.

The three-pole BPF is designed based on open-ended quarter wavelength transmission line resonators. The lengths of the resonators R_1_, R_2_ and R_3_ are L_1_, L_2_ and L_3_, respectively, and the gap between them is G. The 50 Ω input and output transmission lines connected to the first the third resonators produce the required input and output coupling routes and can be calculated by

(1) $$M_{12}=M_{23}=\frac{\mathrm{FBW}}{\sqrt{g_{1}g_{2}}}$$

and the external quality factors can be given by

(2) $$Q_{e1}=Q_{e2}=\frac{g_{0}g_{1}}{\mathrm{FBW}}=\frac{g_{2}g_{3}}{\mathrm{FBW}}$$

**Figure 3:**
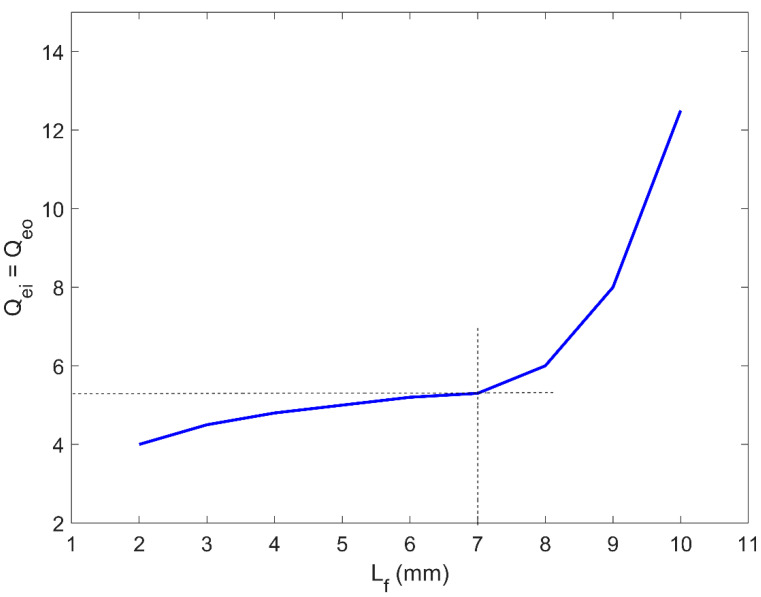
Theoretically calculated input/output external quality factor of the presented three-pole planar BPF.

**Figure 4:**
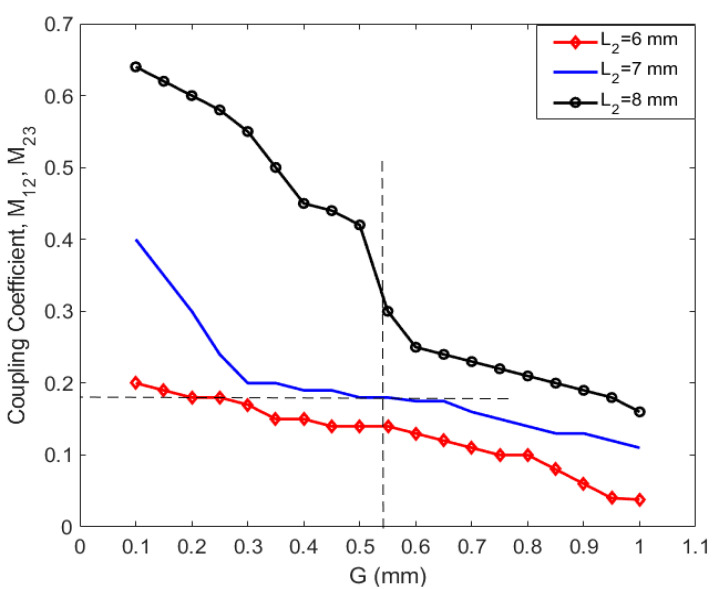
Theoretically calculated coupling coefficient performance of the presented three-pole planar BPF.

These equations are obtained using the low-pass prototype filter properties, where FBW represents the fractional bandwidth, and $g_{0},g_{1},g_{2}$ and $g_{3}$ are the Chebyshev low-pass filter prototype characteristics used to obtain the external quality factor. The external quality factors are calculated and then compared to the value achieved by Equation (3) and by using the CST simulation software. The achieved parameters through different configurations of the microstrip structure are determined and shown in Figure 3 and Figure 4. As seen in Figure 3, it should be noted that the input external quality factor is equal to the output external quality factor, since the geometry of the presented three-pole planar filter is symmetrical on both terminals of the input and output ports.

(3) $$Q_{ei}=Q_{eo}=\frac{f_{0}}{\Delta f_{-3dB}}$$

The transmission lines (R1 and R3) are excited and designed at a specific feeding point with a distance from the open-end (Lf) of 7 mm, to obtain the best value for Q_ei_ and Q_eo_ of the two-port network. The lengths of the resonator R_1_ and R_3_ are 12 mm and the width is 1.1 mm, while the length for the resonator R_2_ is 7.2 mm, and the width is 0.6 mm. The width of the resonators (W_f_) is 1.78 mm, where it is designed to match the 50 Ω impedance at both ports/ends. The gap between the adjacent transmission lines is determined to be 0.55 mm with the optimum coupling coefficients, which provides about 100 MHz impedance bandwidth for the presented planar BPF.

**Figure 5:**
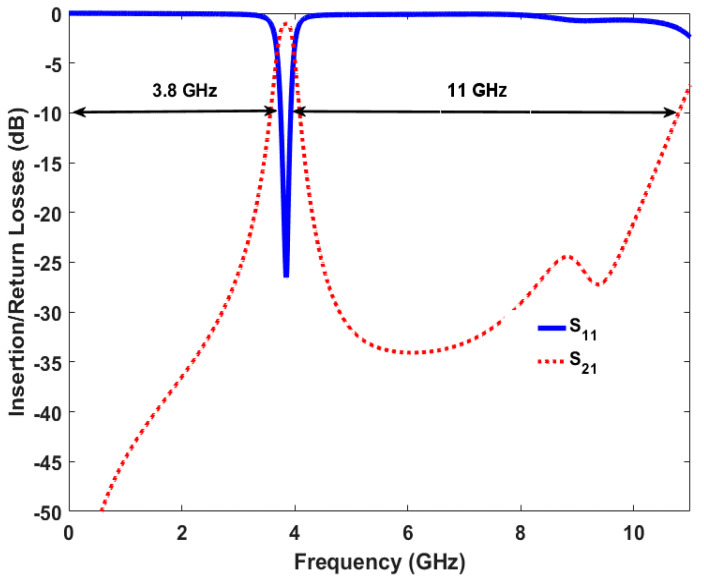
Simulated insertion and return losses for the basic three-pole planar BPF design.

The frequency response for the three-pole BPF is represented in Figure 5. At the center frequency (3.6 GHz), the filter gains a good return loss of more than 25 dB within the targeted sub-6 GHz 5G band and it includes the 3.6 to 3.7 GHz spectrum with a fractional bandwidth (FBW) of about 4%. At the center frequency, the insertion loss is less than 0.9 dB, as illustrated in Figure 5. Wide-stop bands’ rejections of about 3.85 and 11.5 GHz are obtained for lower and upper sides of the transmission band, respectively, each with an insertion loss of less than 15 dB. The roll-off skirts rejection of the transmission band can be enhanced by increasing the number of poles for higher-order designs, although the size of the geometry and the losses will be increased.

**Figure 6:**
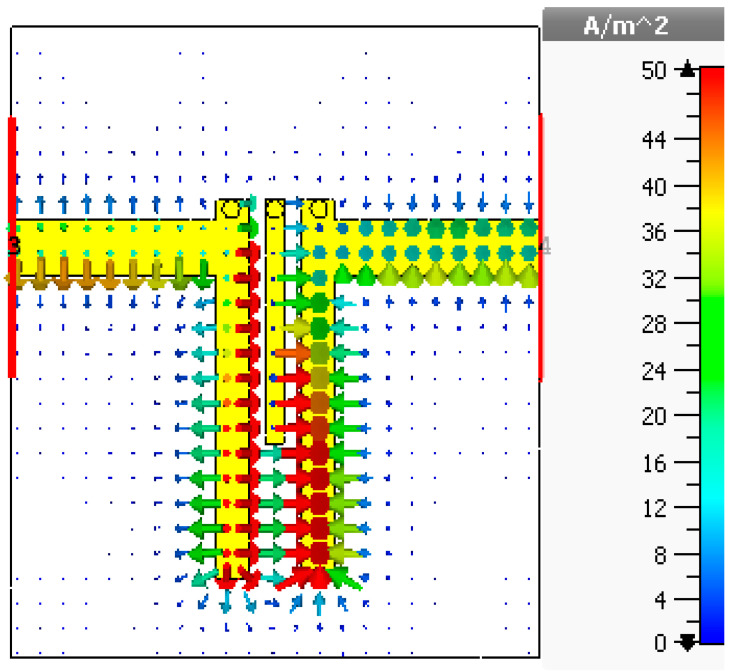
Simulated surface field analysis for the basic three-pole planar BPF design.

Figure 6 shows the electric field density of the asymmetrical coupled line BPF at 3.7 GHz, which is achieved by the CST simulator. The surface current is mainly focused on the first and third transmission line resonators (R_1_ and R_3_), with a maximum field distribution of about 48 A/m^2^ at the edges of the resonators. The next section elaborates on updating the presented basic design by integrating it with two reverse-biased diodes and a suitable DC bias source to provide a reconfigurable performance.

## 3. Reconfigurable BPF Design Using a Hybrid Simulation Technique

**Figure 7:**
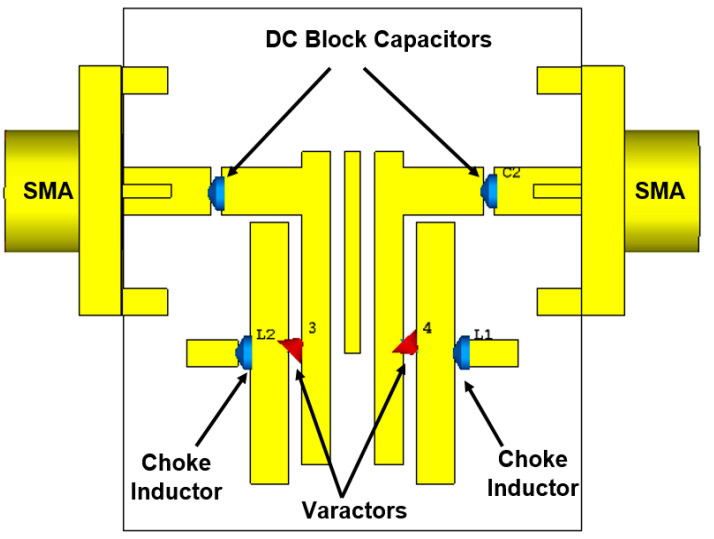
The proposed reconfigurable microstrip planar BPF configuration with DC biasing circuit elements.

**Figure 8:**
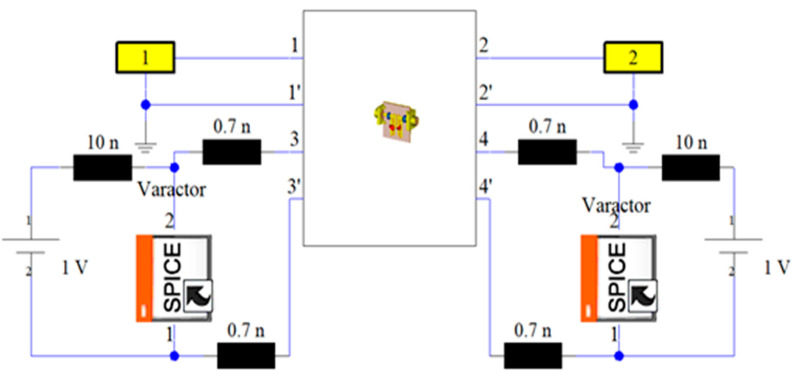
Integrating the RF reconfigurable microstrip filter with the DC circuit and the SPICE representation for the varactors.

Frequency reconfigurability (tenability) is an attractive feature for multi-band wireless communications to deal with different performance variations at the RF front end. The use of varactor diodes is a well-known method for planar tunable BPFs. Figure 7 presents the structure of the designed reconfigurable filter as well as the biasing circuit necessary for tuning the diodes and two RF choke inductors (L_1_ and L_2_) to stop the RF signal going through the DC circuit. Two DC block capacitors (C_1_ and C_2_) are used to protect the VNA from the DC current component. To realize reconfigurable characteristics, two parasitic transmission lines are loaded to the microstrip asymmetrical coupled line BPF presented in the previous section. The dimensions of the parasitic lines are chosen and optimized with a length of 6.5 mm and a width of 1.5 mm, and the gap between these newly added transmission lines and the coupled-line resonators of the basic design is 0.48 mm. The optimized dimensions of the proposed reconfigurable filter are obtained using CST software. Furthermore, to consider all the varactor diode specifications as represented by the datasheet of the manufacturer, EM-circuit simulation between CST MWS and CST DS is also applied and implemented for the introduced tunable planar BPF and illustrated in Figure 8. The varactor diodes are modeled using the SPICE blocks. The terminals 1-1′ and 2-2′ represent the input and output RF feeding ports, while the terminals 3-3′ and 4-4′ are connected to the varactor diodes 1 and 2, respectively.

**Figure 9:**
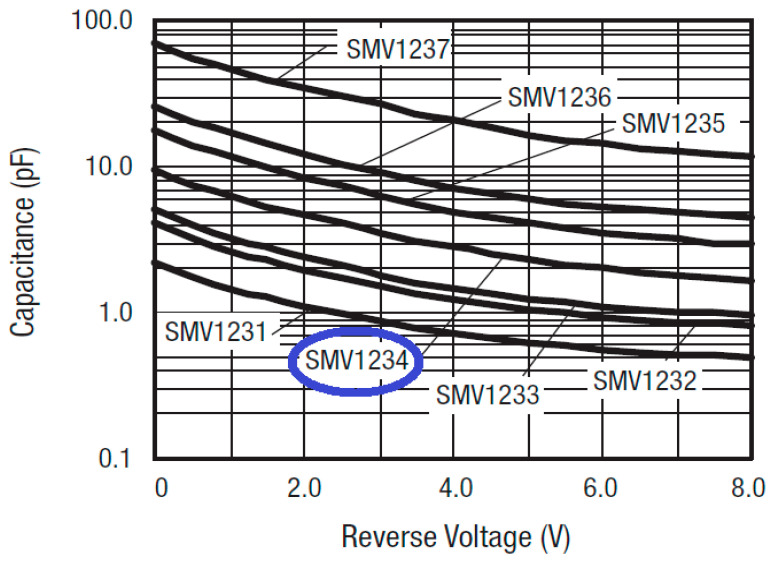
Theoretical reverse biasing voltage performance for several models of Skyworks varactors.

**Figure 10:**
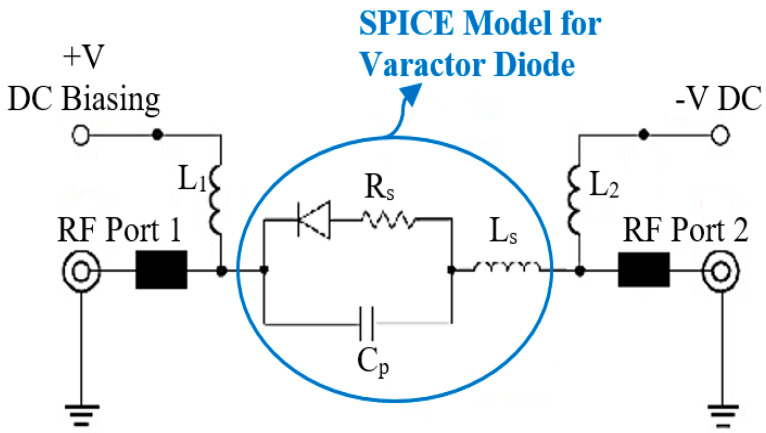
RF and DC biasing circuit for the presented reconfigurable filter.

As seen in Figure 8, the simulation model is also considering the SPICE characteristics for the diode switches and the DC biasing circuit components. Due to the packaging effect, the manufacturer-indicated parasitic inductance is also represented with 0.7 nH inductor for each diode. The two inductors (L_1_ = L_2_ = 10 nH) are used as radio frequency (RF) chokes to suppress the RF leakage into the biasing circuit. CST, based on the time-domain solver, is applied, with ten lines per wavelength as a mesh density, to design and optimize the proposed reconfigurable model to obtain a very compact size and a good s-parameter performance over the entire tuning range. The varactor diodes can tune both the center frequency and impedance bandwidth. Among different varactor models from Skyworks Inc, this filter uses the SMV1234 varactor model with a packaging size of 1.5 × 0.7 mm^2^, since it provides the required range of the reverse biasing capacitor for the proposed filter. Increasing the reverse biasing voltage (VR) will widen the depletion region of the diode, and then, the capacitance value will be decreased, and vice versa, as seen in Figure 9. The reconfigurable microstrip BPF DC circuit with the SPICE representation for the diode switches is represented, as illustrated in Figure 10. It is worthy to say that, by changing the biasing voltage across the practical varactor diodes, the resistor values of the equivalent circuit will also be altered. Thus, it can participate in the entire resonator circuit, leading it to modify the resonating frequency [75].

## 4. Reconfigurable Simulation and Measurement Results

**Figure 11:**
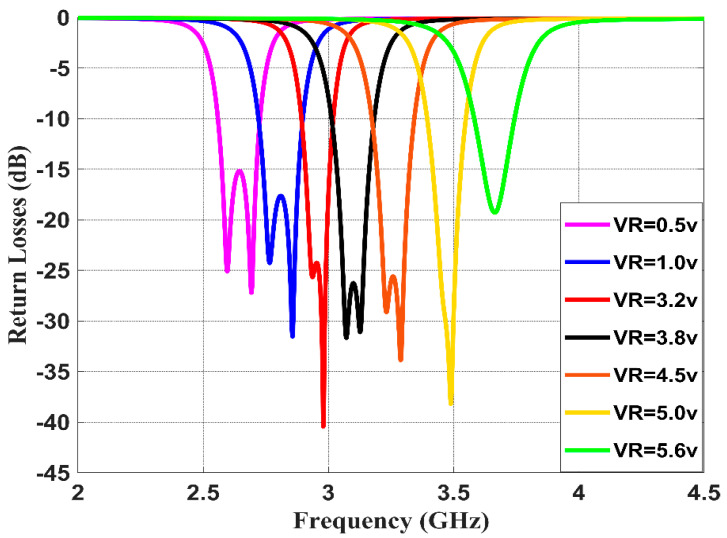
Simulated return losses with different DC reverse bias voltages for the presented reconfigurable filter.

**Figure 12:**
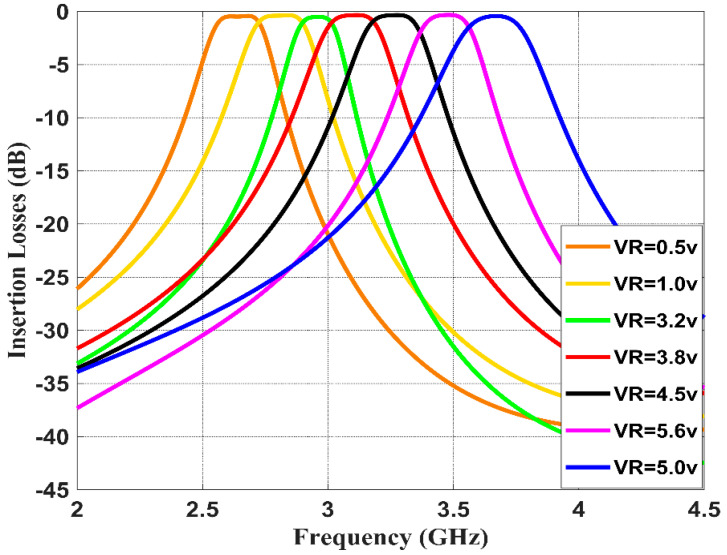
Simulated insertion losses with different DC reverse bias voltages for the presented reconfigurable filter.

This section studies and discusses the simulated and measured return/insertion losses for the designed reconfigurable BPF. The obtained performance for the simulated return losses (shown in Figure 11) shows that increasing the reverse bias voltages across the varactor diode from 0.5 to 5.6 V will increase the capacitance from 2.7 to 8 pF, and thus alters the resonance frequency from 2.5 to 3.8 GHz with insertion losses varying between 15 to 30 dB. The tuning range of the resonance frequency is around 1.3 GHz and the impedance bandwidth is tunable between 95 and 115 MHz. Figure 12 illustrates the simulation insertion loss for the values corresponding to those of Figure 11. A very small insertion loss is observed throughout the entire reconfigurable frequency, which is less than 0.8 dB at the passband. Despite that, the introduced BPF is tunable with the center frequency; it is found that the bandwidth is slightly affected in the range from 95 to 115 MHz.

**Figure 13:**
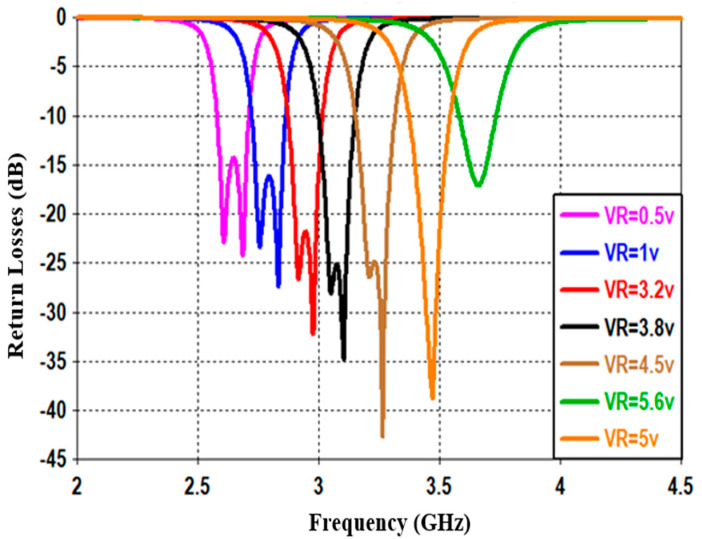
Measured return losses with different DC reverse bias voltages for the presented reconfigurable filter.

**Figure 14:**
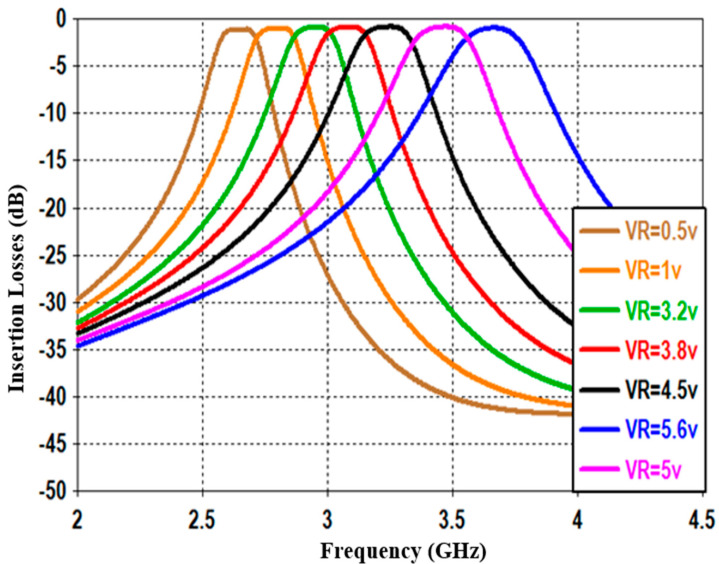
Measured insertion losses with different DC reverse bias voltages for the presented reconfigurable filter.

**Figure 15:**
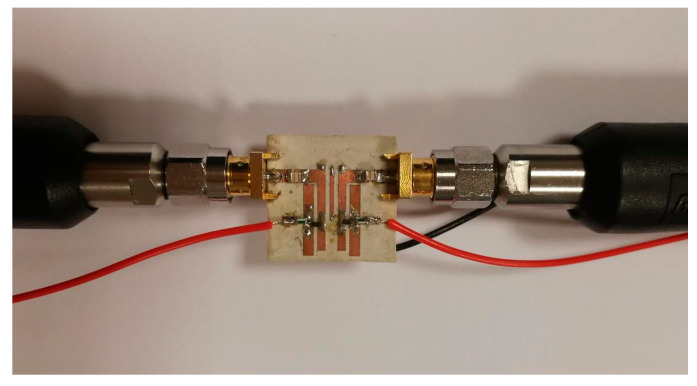
Hardware realization of the fabricated prototype for the proposed reconfigurable filter.

Figure 13 and Figure 14 show the measured return and insertion losses, respectively, for the proposed tunable filter. The s-parameter characteristics are obtained using the HP8510C vector network analyzer (VNA). It should be noted that a very good agreement is achieved between the simulation and measurement results, and this is obtained by using the co-simulation CST MWS and CST DS technique, which has considered all the practical specifications for the varactor diodes and the DC biasing circuit, as explained in the previous section. As seen in Figure 14, the measured insertion losses within the passband are smaller than 1 dB, and the impedance bandwidth varies between 95 and 115 MHz. Figure 15 shows a prototype photograph of the hardware realization for the printed reconfigurable filter, which is used to obtain the measured s-parameters.

**Figure 16:**
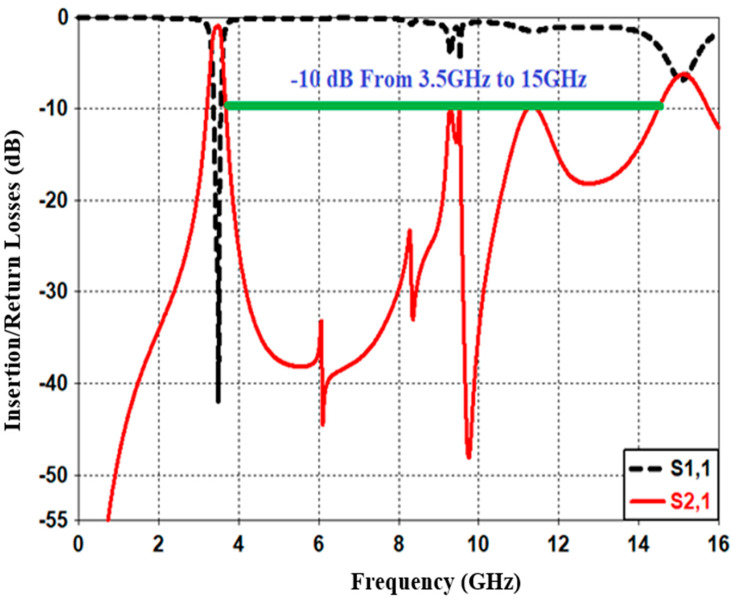
Simulated wide-band s-parameter characteristics for the presented reconfigurable microstrip BPF.

**Figure 17:**
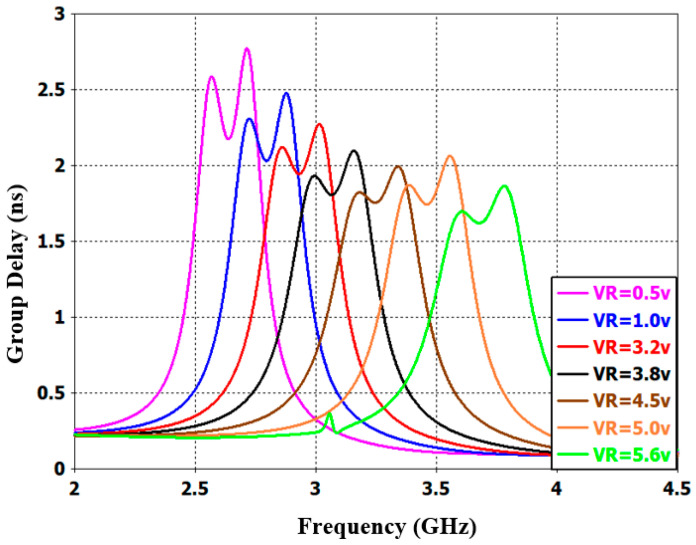
Simulated group delay performance with different DC reverse biasing voltages for the presented reconfigurable microstrip BPF.

**Figure 18:**
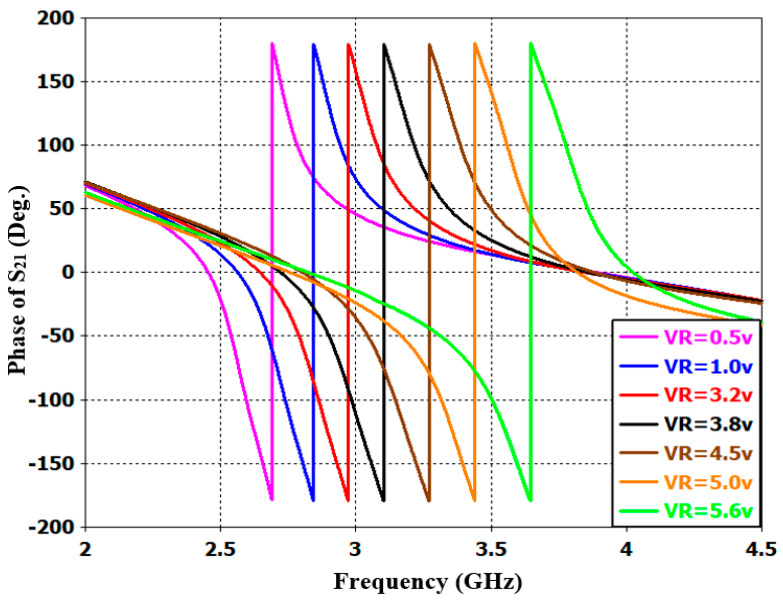
Simulated phase characteristics of S_21_ with different DC reverse bias voltages for the presented reconfigurable microstrip BPF.

Furthermore, Figure 16 shows the wide-band s-parameter response for the introduced reconfigurable microstrip filter over the frequency band from 0 to 16 GHz. The results show good stopband performance for both lower and upper stop-bands of the transmission frequency. Better than 10 dB out-of-band rejection was obtained on the upper side of the transmission frequency with more than 10 dB band. In addition to the infinite transmission zero, three finite transmission zeros are successfully created on the upper stopband bandwidth. The three transmission zeros are located at 6, 8.2, and 8.9 GHz; this produces a good out-of-band and roll-off skirt rejection. The group delay and the phase of S_21_ characteristics are presented in Figure 17 and Figure 18, respectively. It is shown that the filter has a group delay that varies between 1.8 and 2.6 nS over the tuned center frequency. The presented group delay can be controlled by adjusting the S-parameter characteristics, which mainly depend on the selected RF filter configuration. The presented filter has a linear phase of S_21_, which is maintained on 178 degrees over the frequency range and for different values of reverse biasing voltages.

It should be noted that with the rapid development of current 4G and 5G applications, a compact, efficient and reconfigurable planar filter with a wide tuning range is in huge demand. Designing a tunable filter that covers both 4G and 5G spectrum using the same configuration will also be required for several wireless applications. According to what is shown, the proposed tunable filter can offer these requirements, where the design has a very compact size and operates at a centre frequency range from 2.5 GHz (4G) to 3.8 GHz (5G) with a very low insertion loss of 0.8 dB. Moreover, Table 1 shows the comparative performance of the presented reconfigurable microstrip BPF with other, similar designs from the literature. It is shown that the reported filter has a wider tuning range and wider impedance bandwidth, smaller insertion losses and a smaller size compared to the designs presented in [56,57,59,60,61,62]. The tunable filters presented in [60,61] have an impedance bandwidth of only 40 MHz. The presented tunable filter and the filter introduced in [62] use only two varactor diode switches and a simple basing circuit to achieve the tunable frequency characteristics. As a result, the filter presented in this work has a relatively very good performance in terms of return/insertion losses, group delay and the phase of S_21_, as well as other features such as a compact size, a few tuning diodes and simple structure.

## 5. Conclusions

A new and very compact tunable BPF with a wide reconfigurable s-parameter based on a new EM-circuit co-simulation scheme is presented and discussed in this paper. A CST simulator is utilized to design and optimize the presented filter. An integrated model between CST MWS and CST DS is utilized to design and construct the RF and DC circuits. This presented model is considered the SPICE representation for the varactor switches as well as the packaging effect of the electronic components. The proposed BPF is tunable in the frequency range from 2.5 to 3.8 GHz by using only two varactor diode switches and a simple biasing circuit. The proposed reconfigurable microstrip BPF has several attractive features, such as a very compact and simple design, wide tuning range, high stopband rejection, and a very low insertion loss. Measurement results have been presented, showing interesting performance and an excellent agreement with the simulation results. The presented reconfigurable filter, covering a wide tunable s-parameter range, considers both the 4G and sub-6 GHz 5G spectrum and can be a good candidate for present and future RF systems.

## Figures and Tables

**Table 1:** Performance comparison with some other tunable BPFs.

Ref.	Tuning Range (GHz)/(%)	Fractional Tuning Range (%)	BW (MHz)/(%)	Tunable Fractional Bandwidth (%)	No. of Switches	IL (dB)	$\mathrm{Filter}\;\mathrm{Size}({\mathrm{mm}}^{3})/(\lambda_{g}^{3})$
[56]	0.6–1.0	50	85–95	8–9	3	2.2	30 × 23 × 1.27/ 0.2 × 0.15 × 0.008
[57]	1.5–2.0	28.5	93–110	5–7	4	4	36 × 30 × 0.80/ 0.60 × 0.50 × 0.013
[59]	0.66–0.99	41	92–108	6–8	4	0.75	72 × 70 × 1.6/ 0.53 × 0.53 × 0.01
[60]	1.1–2.1	62	28–40	1–3	7	6	52 × 12 × 1.6/ 0.61 × 0.14 × 0.02
[61]	1.7–2.9	52.1	32–40	1–2	7	4	36 × 35 × 0.8/ 0.65 × 0.63 × 0.02
[62]	0.76–2	69.2	75–150	1–7	4	1.2	100 × 8 × 0.50/ 0.80 × 0.06 × 0.004
[63]	0.97–1.72	55	56–64	3–5	4	4.5	42 × 35 × 0.63/ 0.43 × 0.36 × 0.006
[64]	0.90–1.7	61.5	80–85	4–6	4	4.3	48 × 40 × 0.51/ 0.46 × 0.40 × 0.005
this work	2.5–3.8	41.3	95–115	7–9	2	0.8	13 × 8 × 0.80/ 0.3 × 0.2 × 0.02

IL: Insertion loss, λ_g_: Guided wavelength at the centre frequency.
